# Production of ginsenoside compound K from American ginseng extract by fed-batch culture of *Aspergillus tubingensis*

**DOI:** 10.1186/s13568-023-01556-5

**Published:** 2023-06-25

**Authors:** Woo-Seok Song, Kyung-Chul Shin, Deok-Kun Oh

**Affiliations:** 1 Department of Bioscience and Biotechnology, Konkuk University, 120 Neungdong-Ro, Gwangjin-Gu, Seoul, 05029 Republic of Korea; 2 Department of Integrative Bioscience and Biotechnology, Konkuk University, 120 Neungdong-Ro, Gwangjin-Gu, Seoul, 05029 Republic of Korea

**Keywords:** *Aspergillus tubingensis*, Compound K, Feed optimization, Fed-batch fermentation, American ginseng extract, Protopanaxadiol-type ginsenosides

## Abstract

Compound K (C-K), one of the most bioactive ginsenoside, is produced by hydrolyzing the glycoside moieties of protopanaxadiol (PPD)-type glycosylated ginsenosides in the ginseng extract. To enhance the biotransformation of PPD-type ginsenosides in American ginseng extract (AGE) into C-K, the optimization of the feed type, concentration, and period for the carbon source sucrose and the reactant AGE was performed in fed-batch fermentation of *Aspergillus tubingensis* using a fermenter*.* The concentration (3.94 g/L) and productivity (27.4 mg/L/h) of C-K after feed optimization in fed-batch fermentation increased 3.1-fold compared to those (1.29 g/L and 8.96 mg/L/h) in batch fermentation, and a molar conversion of 100% was achieved. To the best of our knowledge, this is the first trial of fed-batch fermentation to convert ginseng extract into deglycosylated ginsenoside and the highest reported C-K concentration and productivity using ginseng extract via fermentation. After ethanol and resin treatments, C-K solids with purities of 59% and 96% were obtained from the fermentation broth as food- and pharmaceutical-grade products, respectively.

**Graphical Abstract:**

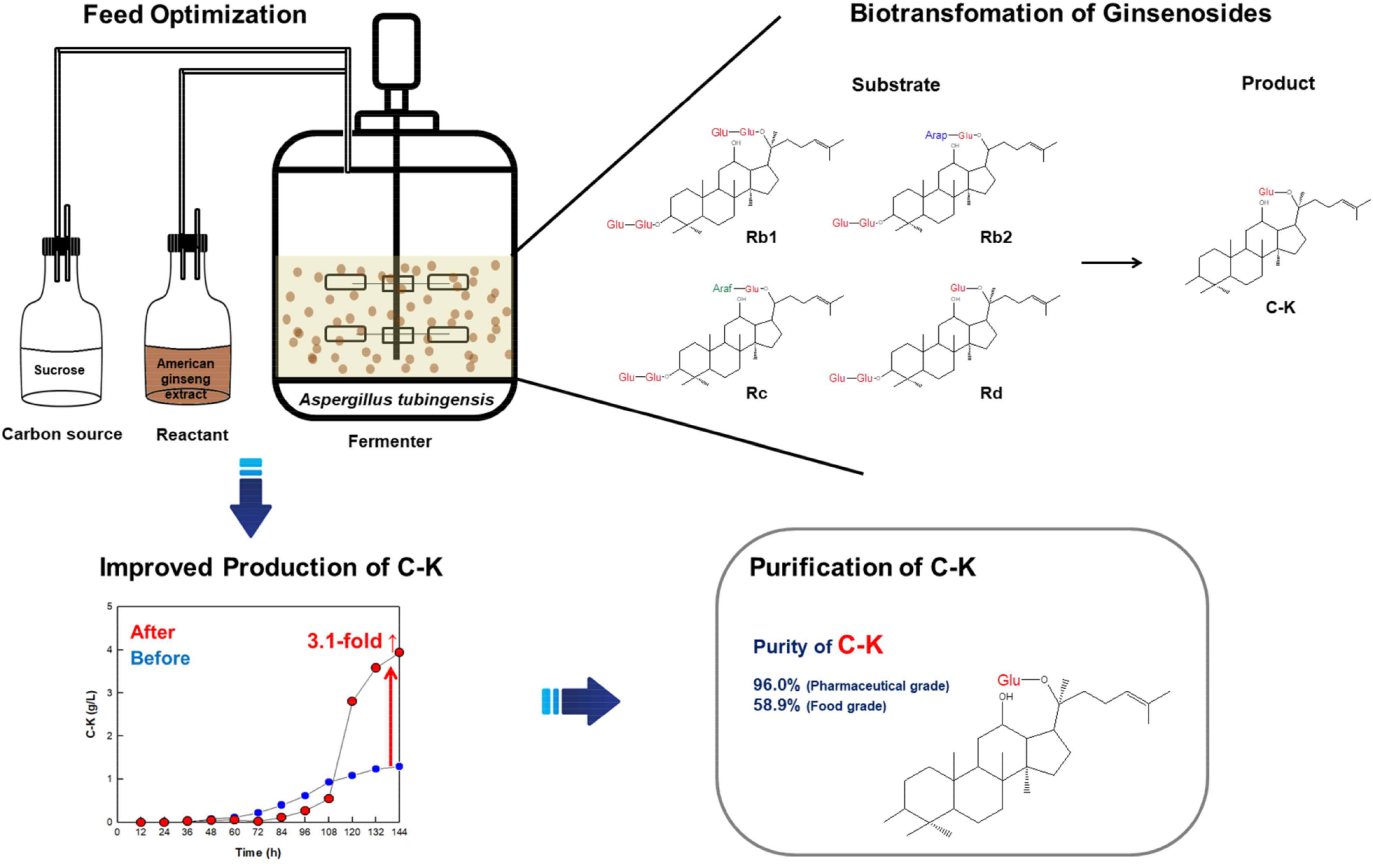

**Supplementary Information:**

The online version contains supplementary material available at 10.1186/s13568-023-01556-5.

## Key points

- This is the first fed-batch fermentation for biotransformation of ginseng extract
- The highest production of compound K via fermentation are achieved
- This is the first report on the purification of compound K from fermentation broth

**Supplementary Information:**

The online version contains supplementary material available at 10.1186/s13568-023-01556-5.

## Introduction

Ginseng (*Panax ginseng* C.A. Meyer), a traditional herbal medicine, is used in East Asia to maintain physical vitality and strengthen immunity (Lieberman 2001). Ginsenosides, which are the principally active compounds in ginseng, are divided into protopanaxadiol (PPD)- and protopanaxatriol (PPT)-type ginsenosides according to the number and position of the hydroxyl groups attached to the triterpenoid aglycone (Park et al. 2010). They contain glycosides, such as d-xylopyranoside, d-glucopyranoside, l-rhamnopyranoside, l-arabinopyranoside, and l-arabinofuranoside, which are attached to C-3, C-6, and C-20 in PPD- and PPT-type aglycones via glycosidic bonds.

Major ginsenosides, such as Rb1, Rb2, Rc, Rd, Re, and Rf, contain 2–4 glycoside molecules, whereas minor ginsenosides, such as Rh1, Rh2, F2, Rg3, and compound K (C-K), which are deglycosylated from major ginsenosides, consist of 1–2 glycoside molecules (Yue et al. 2021). Minor ginsenosides, including C-K, exhibit higher bioactivity than major ginsenosides owing to their better permeability across cell membranes in the gastrointestinal tract (Park et al. 2010). Thus, major ginsenosides must be converted into minor ginsenosides via the hydrolysis of glycoside moieties in ginsenosides.

C-K, 20-*O*-β-d-glucopyranosyl-20(*S*)-PPD, is the most extensively studied and one of the most bioactive ginsenoside. The market demand for C-K in the food, cosmetic, and pharmaceutical industries is rapidly increasing because of its excellent pharmacological activities, (Liu et al. 2022) including anti-allergic (Shin et al. 2005), anti-cancer (Yin et al. 2021), anti-diabetic (Li et al. 2012), anti-fatigue (Yang et al. 2015), anti-inflammatory (Hossen et al. 2017), anti-oxidative (Hossen et al. 2017), anti-photoaging (Hong et al. 2018), anti-wrinkling (Lim et al. 2015), hepatoprotective (Igami et al. 2015), and skin protective effects (Kim et al. 2018).

**Fig. 1:**
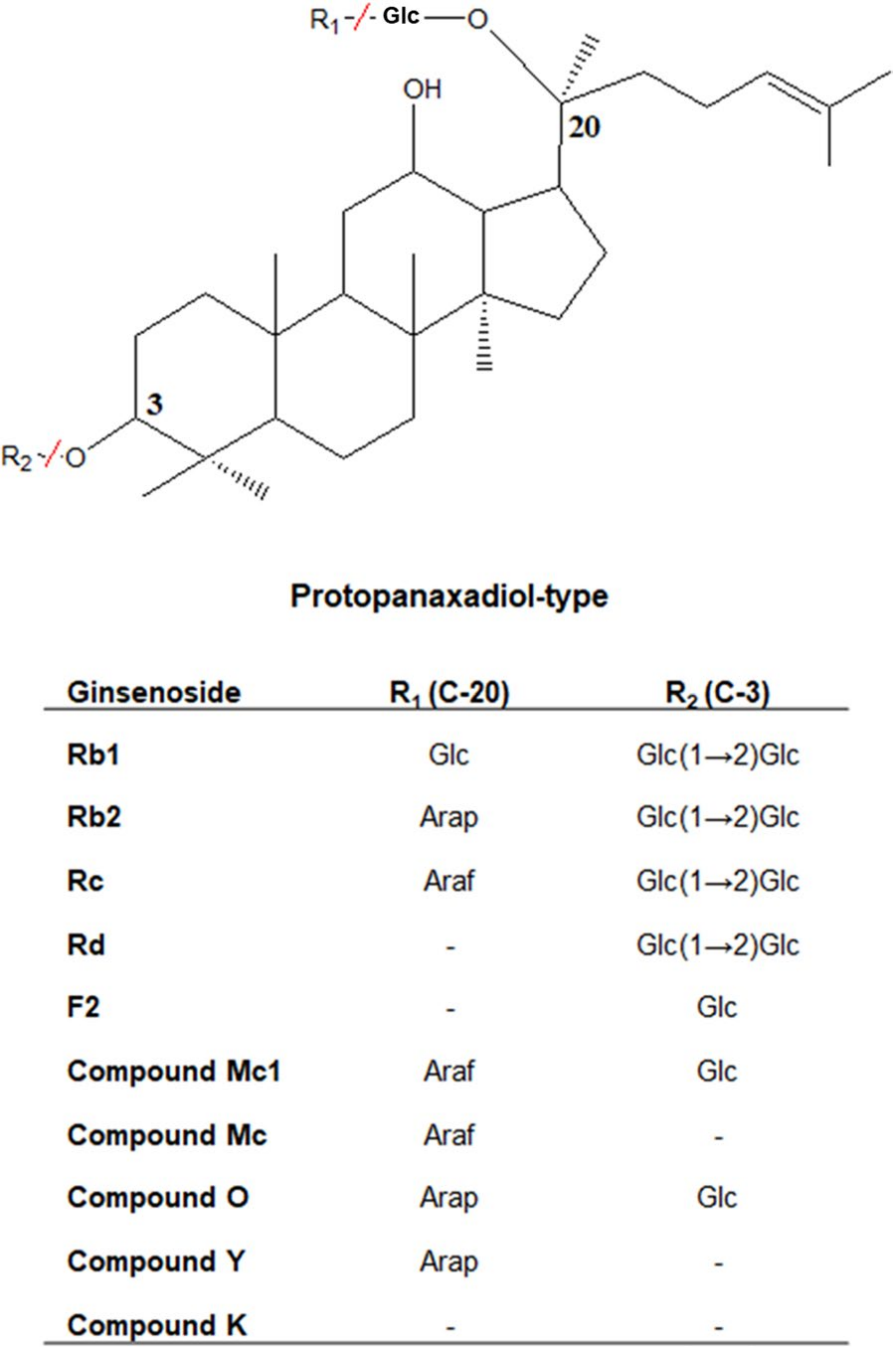
Chemical structures of protopanaxadiol (PPD)-type ginsenosides involved in the biotransformation of ginsenosides Rb1, Rb2, Rc, and Rd in the ginseng extract into compound K (C-K) by *Aspergillus tubingensis*. Glc, *β-*d-glucopyranoside; Araf, *α*-l-arabinofuranoside; and Arap, α-l-arabinopyranoside. Ginsenosides F2, compound Mc1 (C-Mc1), compound O (C-O), compound Mc (C-Mc), and compound Y (C-Y) were the intermediates

Chemical, physical, and biological methods have been used to produce C-K with one inner glucoside at C-20 from PPD-type major ginsenosides, including Rb1, Rb2, Rc, and Rd, in ginseng extracts by hydrolyzing glycoside moieties (Fig. 1) (Zheng et al. 2017). However, physical and chemical methods have some limitations in producing C-K because of their low selectivity, by-product formation, generation of environmental pollution, and/or high energy requirements. To overcome these limitations, biological methods, such as enzyme and cell transformations and fermentation, have been widely used for C-K production (Kim et al. 2021, 2019; Zhou et al. 2008). Although enzyme and cell transformations show higher conversion and productivity than those by fermentation, fermentation is the most cost-effective method because it does not require additional cultivation for enzyme or cell preparation and enzyme purification or cell harvest process, and it can use both intracellular and extracellular enzymes (Jeong et al. 2020; Song et al. 2022). However, fermentation has resulted in a lower concentration and productivity of C-K (Bae et al. 2011; Han et al. 2007; Hsu et al. 2013; Li et al. 2016; Li and Ji 2017; Zhou et al. 2008). To overcome the problems, fed-batch fermentation of carbon source and/or reactant is required.

In this study, we performed fed-batch fermentation of the generally recognized as safe (GRAS) fungus *Aspergillus tubingensis* using a fermenter for the enhanced biotransformation of the American ginseng extract (AGE) into C-K by optimizing the feed type, concentration, and period for sucrose as a carbon source and AGE as a reactant. C-K was then purified from the fermentation broth for food and pharmaceutical applications.

## Materials and methods

### Materials

The ginsenoside standards (≥ 98% purity), including Rb1, Rb2, Rc, Rd, F2, compound Mc1 (C-Mc1), compound Mc (C-Mc), compound O (C-O), compound Y (C-Y), and C-K, were purchased from Ambo Institute (Seoul, Republic of Korea). AGE purchased from Ace EMzyme (Ansung, Republic of Korea) contained 38.5% (w/w) PPD-type ginsenosides, which consisted of 20.4% Rb1, 1.2% Rb2, 7.4% Rc, and 9.5% (w/w) Rd. A sucrose assay kit was purchased from BioAssay Systems (Hayward, CA, USA).

### Fungal strain and fermentation media

*A. tubingensis* Korean Collection for Type Cultures (KCTC) 14,166 (Daejeon, Republic of Korea) was used for C-K production. The inoculum preparation and growth medium for *A. tubingensis* were the same as those described previously (Song et al. 2022). The optimal carbon and nitrogen sources for C-K production were sucrose and soy protein concentrate, respectively. Therefore, fermentation medium contained 20 g/L sucrose, 10 g/L soy protein concentrate, 2 g/L rice straw, 5 g/L KH_2_PO_4_, 5 g/L Na_2_HPO_4_, 0.3 g/L CaCl_2_, 0.3 g/L MgSO_4_·7H_2_O, 5 mg/L, FeSO_4_·7H_2_O, and 1.3 mg/L MnSO_4_·H_2_O, and the pH and temperature during fermentation were adjusted to 5.0 and 28 °C, respectively.

### Fermentation conditions

The mycelia were incubated in a 250-mL baffled flask containing 50 mL of the fermentation medium at 28 °C for 24 h. The grown mycelia were transferred into a 3-L fermenter (Mardo-05D-PB; BioCNS, Daejeon, Republic of Korea) with 1 L of fermentation medium. The fermentation broth was incubated at 28 °C and pH 5.0 for 144 − 168 h. The pH was maintained at a constant value by adjusting with 15% phosphoric acid and ammonia solutions during fermentation. The agitation speed was adjusted from 150 to 1000 rpm to maintain the dissolved oxygen levels above 20%, and the aeration rate was 1 L/min. In fed-batch fermentation, 180 mLsucrose and 120 mL AGE were intermittently or continuously added to the fermenter using a peristatic pump (basic type; Longer Precision Pump, Hebei, China). Samples were harvested from the fermenter at different time points.

### Feed optimization of sucrose

In batch fermentation of *A. tubingensis* using the fermenter, the initial sucrose concentration was varied from 10 to 50 g/L. For pulse feeding of sucrose, 20 g/L sucrose was initially added, and 5 g/L sucrose was later added twice when the residual sucrose level dropped below 5 g/L. For continuous feeding, 20 g/L sucrose was initially added, and then continuous feeding of 10 g/L sucrose was started at 12 h and stopped at time points ranging from 108 to 144 h for determining the optimal feeding-stop time of sucrose addition for C-K production. The total added concentration of sucrose, including the initial 20 g/L sucrose and continuous feeding of 10 − 60 g/L sucrose from 12 to 132 h, was varied from 30 to 80 g/L to determine the optimal total added concentration. For all experiments with sucrose addition, 8 g/L AGE was added twice at 36 and 48 h of fermentation.

### Feed optimization of AGE

The optimal conditions of sucrose feeding for C-K production were the initial addition of 20 g/L sucrose, followed by continuous addition of 40 g/l sucrose from 12 to 132 h at a feeding rate of 0.33 g/L/h. Under these optimal conditions, for pulse feeding, 8 g/L AGE was added twice at 36 and 48 h, and for continuous feeding, AGE was continuously added from 36 to 132 h at a feeding rate of 0.167 g/L/h with a total concentration of 16 g/L. The feeding-start time of AGE for the maximal production of C-K was optimized by varying the time from 6 to 32 h at a feeding-stop time of 132 h, and the feeding-stop time of AGE was optimized by varying the time from 60 to 132 h at a feeding-start time of 12 h.

### C-K production under optimized feed conditions

The effect of the total added concentration of AGE was investigated by varying the concentration from 8 to 32 g/L with continuous feeding from the optimal feeding start time of 12 h to the optimal feeding stop time of 84 h under the optimized conditions of sucrose feeding. The fermentation was terminated at 156 h, when the C-K concentration reached a plateau. AGE at 8 or 20 g/L for the maximal biotransformation or production of C-K was continuously added from 12 to 84 h at a flow rate of 0.11 or 0.28 g/L/h, respectively.

### Determination of dry cell weight, sucrose concentration, and *β*-glucosidase activity

The fermentation broth was filtered through Whatman No. 1 filter paper, washed with distilled water, and the collected mycelia were dried in a dry oven at 105 °C for 12 h. After drying, the dry cell weight was measured in g/L. The filtered solution (100 µL) was used to determine the residual sucrose concentration or β-glucosidase activity, which was measured using a sucrose assay kit or *para*-nitrophenyl β-d-glucopyranoside (*p*NPGlc), respectively. To determine β-glucosidase activity, the hydrolysis reaction was performed at 55 °C in 50 mM citrate/phosphate buffer (pH 4.0) containing 1 mM *p*NPGlc for 10 min and then stopped by adding 2 M Na_2_CO_3_ as the final concentration. The increase in absorbance at 405 nm caused by the release of *para*-nitrophenol (*p*NP) was measured. One unit of β-glucosidase activity was the amount of enzyme that produced 1 μmol of *p*NP per minute.

### Purification of C-K

After fermentation, mycelia were collected as a precipitate via centrifugation at 3000 × *g* for 30 min, and C-K was extracted thrice from the mycelia with 1 L of 100% ethanol at 4 °C for 24 h. The supernatant was dried using an evaporator and extracted with 1 L of 100% ethanol. The residual mycelia in the ethanol extract were removed via centrifugation at 3000 × *g* for 30 min and filtration using a 0.2-μm pore-size filter. The supernatant extract was filtered using the 0.2-μm pore-size filter to remove the precipitate. The extracts were concentrated using an evaporator and dried using a dry oven at 105 °C overnight, and the weights of dried solids containing C-K were measured. All extracts were collected and concentrated using an evaporator followed by ultrafiltration using Centricon (Amicon Ultra-15; Millipore, Burlington, MA, USA) to remove the cell debris. The ultrafiltered product was purified using an octadecyl-silica (ODS) A column (3 × 60 cm; YMC, Kyoto, Japan). The column was eluted with 55% acetonitrile at a flow rate of 10 mL/min to remove other by-products, except for C-K, and eluted with 75% acetonitrile at 10 mL/min to obtain C-K. The fraction of 75% acetonitrile was collected and concentrated using an evaporator. The concentrated C-K was then separated using a preparative high-performance liquid chromatography (Prep-HPLC; Agilent 1260, Santa Clara, CA, USA) system equipped with a Hydrosphere C18 preparative column (10 × 250 mm; YMC). The column was eluted with water at a flow rate of 5.0 mL/min at 30 °C and the eluent was monitored using an ultraviolet detector at an absorbance of 203 nm.

### Analysis of ginsenosides

The fermentation was terminated, and the broth was extracted with n-butanol at a ratio of 1:1. After extraction, 1.0 mg/mL of the internal standard digoxin was added to the extracted solution. The *n*-butanol layer was collected via centrifugation at 13,000 × *g* for 10 min and evaporated to dryness. A same volume of methanol was added to the dried residue. Ginsenosides dissolved in methanol were analyzed using an Agilent 1100 Infinity HPLC system with an ultraviolet detector at 203 nm and an ODS column (YMC). The column was eluted at 40 °C at a flow rate of 1 mL/min with a gradient of acetonitrile/water (v/v) from 30:70 to 60:40 for 20 min, 60:40 to 90:10 for 10 min, 90:10 to 30:70 for 5 min, and 30:70 for 10 min. All PPD-type ginsenosides were quantified using calibration curves with ginsenoside standards.

### Statistical analysis

The means with standard errors for all experiments, including the feed optimization of sucrose and AGE, were calculated in duplicate. One-way analysis of variance was performed using Tukey’s method with a significance level of *p* < 0.05 using SigmaPlot program (10.0 version, Systat Software, Chicago, IL, USA).

## Results

### Effect of initial sucrose concentration on c-k production in batch fermentation of *A. tubingensis*

The effect of the initial sucrose concentration on C-K production was investigated in batch fermentation using a fermenter by varying the concentration from 10 to 50 g/L to determine the optimal initial sucrose concentration for C-K production (Table 1). Initial sucrose concentrations above 20 g/L resulted in decreased concentration, productivity, and molar conversion of C-K from AGE. With increasing sucrose concentrations above 20 g/L, the activity of extracellular β-glucosidase for *p*NPGlc decreased, suggesting that concentrations above 20 g/L sucrose inhibited the formation of C-K-producing enzymes. At 20 g/L sucrose, *A. tubingensis* produced 1.29 g/L C-K for 144 h, with a productivity of 8.96 mg/L/h, and a molar conversion of 33.6% from PPD-type ginsenosides in AGE. The concentration, productivity, and molar conversion were 1.3-fold higher than those at 10 g/L sucrose and 1.6-, 1.7, and 1.6-fold higher than those at 30 g/L sucrose, respectively. These results indicated that the optimal initial sucrose concentration was 20 g/L.

**Table 1:** Effect of initial sucrose concentration on compound K (C-K) production in batch fermentation of *Aspergillus tubingensis*

Initial sucrose (g/L)	X_max_ (g/L)	μ_max_ (1/h)	Activity^a^ (U/mL)	C-K_max_ (g/L)	*Y*_P/S_ (g/g)	*Y*_P/AGE_ (g/g)	q_P_ (mg/L/h)	Molar conversion (%)	*f*_t_ (h)
10	18.6 ± 0.2	0.31 ± 0.003	5.64 ± 0.05	0.99 ± 0.01	0.09 ± 0.001	0.06 ± 0.001	6.88 ± 0.06	25.7 ± 0.26	144
20	19.8 ± 0.3	0.33 ± 0.006	6.43 ± 0.04	1.29 ± 0.02	0.07 ± 0.001	0.08 ± 0.001	8.96 ± 0.10	33.6 ± 0.39	144
30	23.7 ± 0.4	0.33 ± 0.006	3.65 ± 0.04	0.83 ± 0.02	0.03 ± 0.001	0.05 ± 0.001	5.32 ± 0.13	21.6 ± 0.52	156
40	31.2 ± 0.5	0.52 ± 0.004	1.76 ± 0.05	0.71 ± 0.02	0.02 ± 0.001	0.04 ± 0.001	4.55 ± 0.09	18.5 ± 0.39	156
50	26.4 ± 0.3	0.44 ± 0.006	0.94 ± 0.04	0.29 ± 0.02	0.01 ± 0.001	0.02 ± 0.001	1.86 ± 0.02	7.55 ± 0.65	156

μ_max_: maximum specific growth rate, *Y*_P/S_: C-K yield from sucrose, *Y*_P/AGE_: C-K yield from AGE, q_P_: volumetric production rate of C-K, *f*_t_: fermentation time

^a^β-Glucosidase activity was measured by the release of *para*-nitrophenol (*p*NP) from *para*-nitrophenyl β-d-glucopyranoside (*p*NPGlc)

### Comparisons of the initial, pulse, and continuous feedings of sucrose in batch and fed-batch fermentations of *A. tubingensis*.

Three feeding types, including the initial feeding of sucrose in batch fermentation and pulse and continuous feedings of sucrose in fed-batch fermentation, were compared to determine the optimal feeding type of sucrose for C-K production. In flask fermentation, 8 g/L AGE was added twice at 48 and 60 h of fermentation for the maximal cell concentration at 72 h.(Song et al. 2022) In fermenter fermentation for sucrose feeding, the feeding time points of 8 g/L AGE were changed to 36 and 48 h because the time required to reach the maximal cell concentration was reduced to 60 h as 12 h decrease compared to that of flask fermentation.

**Fig. 2:**
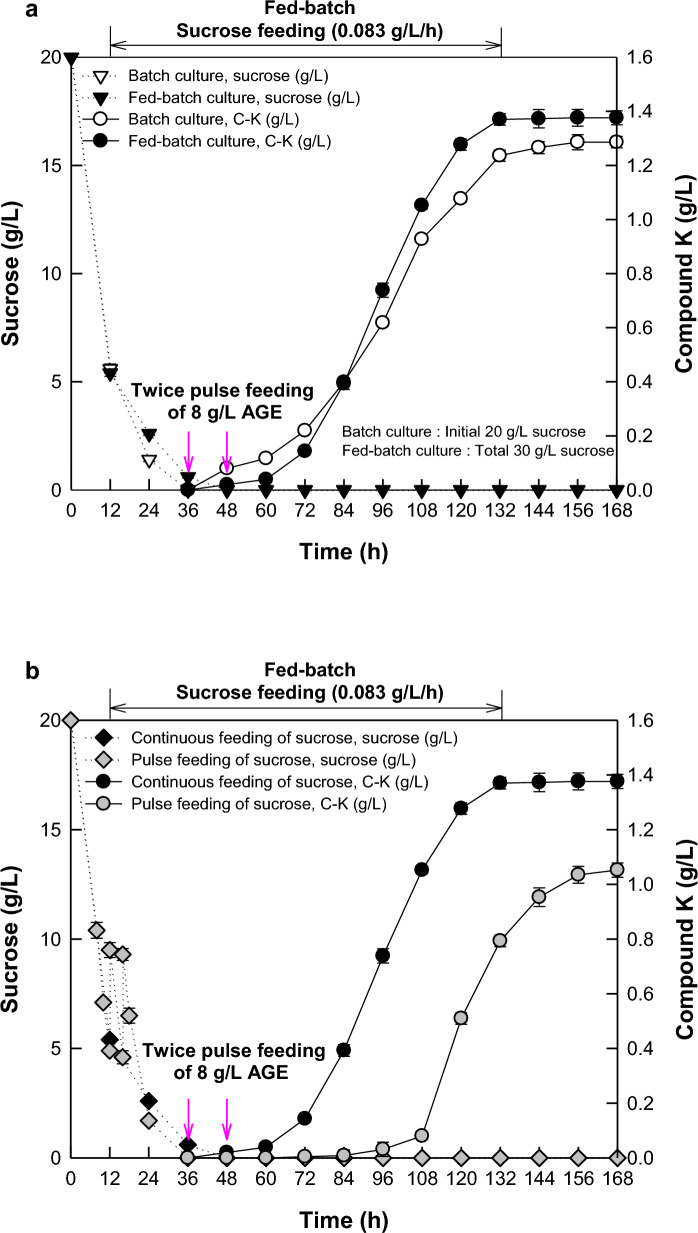
Comparison of the feeding types of sucrose for C-K production by *A. tubingensis*. **a** Comparison of the initial feeding of sucrose in batch fermentation with continuous feeding in fed-batch fermentation. **b** Comparison of pulse and continuous feedings of sucrose in fed-batch fermentation. For initial feeding, 20 g/L sucrose was initially added. For pulse feeding, 20 g/L sucrose was initially added, and 5 g/L sucrose was intermittently added twice when the sucrose concentration dropped below 5 g/L. For continuous feeding, 20 g/L sucrose was initially added, and 10 g/L sucrose was continuously added from 12 to132 h at flow rate of 0.083 g/L/h. AGE at 8 g/L was intermittently added twice at 36 and 48 h

After fermentation, the concentration, productivity, and molar conversion of C-K for continuous feeding were 1.38 g/L, 8.15 mg/L/h, and 35.6%, respectively, showing 1.1-fold increases compared to those for initial feeding of 20 g/L sucrose (Fig. 2a), and those for pulse feeding of sucrose reduced to 76.8% compared to those for continuous feeding, respectively (Fig. 2b). When the total added concentration of sucrose was 30 g/L, the concentration, productivity, and molar conversion of C-K for continuous feeding were 1.7-, 1.5-, and 1.6-fold higher than those for initial feeding, respectively (Table 1), indicating that continuous feeding is an optimal feeding type when the total added concentration of sucrose was the same as those of the initial, pulse, and continuous feedings. Thus, sucrose was continuously added to all subsequent fermentations.

### Optimization of continuous feeding of sucrose as a carbon source in fed-batch fermentation of *A. tubingensis*.

**Fig. 3:**
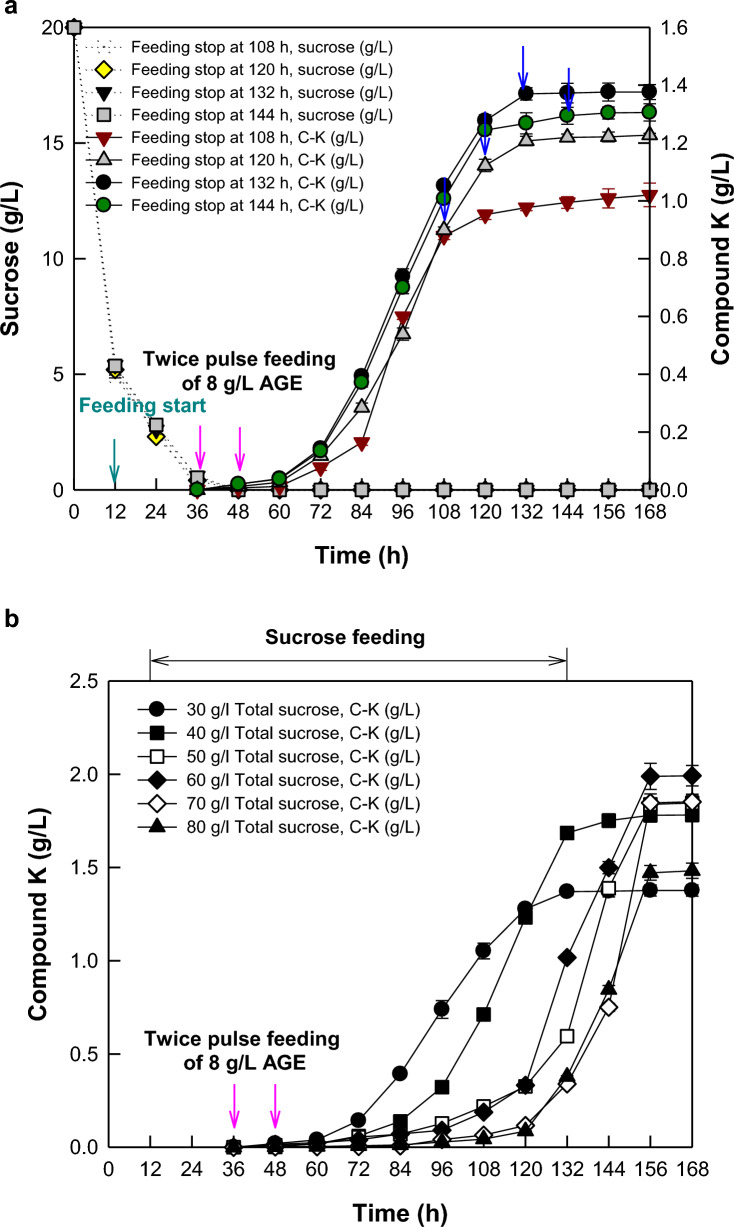
Effects of feeding-stop time and total added concentration of sucrose on C-K production by *A. tubingensis*. **a** Effect of feeding-stop time of sucrose. Sucrose at 20 g/L was initially added, and continuous feeding of 10 g/L sucrose was started at 12 h and stopped at the time from 108 to 144 h. **b** Effect of total added sucrose concentration. Sucrose at 20 g/L was initially added and 10–60 g/L sucrose was continuously added from 12 to 132 h. AGE at 8 g/L was intermittently added twice at 36 and 48 h. Dark cyan and blue arrows indicate the feeding-start and feeding-stop time points of sucrose, respectively

For continuous feeding, feeding of 10 g/L sucrose was started at 12 h, in which the residual sucrose was approximately 5 g/L, and stopped at 108, 120, 132, and 144 h of fermentation. After 48 h, residual concentrations of sucrose were maintained below 0.5 g/L. C-K production was highest at 1.38 g/L when feeding was stopped at 132 h (Fig. 3a). These results indicate that the supply period of sucrose from 12 to 132 h of fermentation time was optimal for the maximal production of C-K.

C-K production in the fed-batch fermentation was investigated by varying the total added concentration of sucrose from 30 to 80 g/L (Fig. 3b). After 48 h, residual concentrations of sucrose were maintained below 1.0 g/L. When the total added concentration of sucrose was 60 g/L, the concentration (1.99 g/L) and productivity (12.8 mg/L/h) of C-K were highest, which were 1.4- and 1.6-fold higher than those at the total added concentration of 30 g/L sucrose, respectively. However, above 60 g/L sucrose, C-K concentration decreased as the total added sucrose concentration increased. These results demonstrated that the optimal total sucrose concentration for C-K production was 60 g/l. For all subsequent AGE feeding, 20 g/L sucrose was initially added, and 40 g/L sucrose was continuously added from 12 to 132 h at a flow rate of 0.33 g/L/h.

### Comparison of pulse and continuous feedings of age as a reactant in fed-batch fermentation of *A. tubingensis*

Pulse and continuous feedings with a total added AGE of 16 g/L for C-K production were compared in fed-batch fermentation (Additional file 1: Fig. S1). The concentration (2.64 g/L) and productivity (16.9 mg/L/h) of C-K by continuous feeding were 1.3-fold higher than those by pulse feeding, respectively. Therefore, AGE was continuously added to all the subsequent fed-batch fermentations.

### Optimization of continuous feeding of age as reactant in fed-batch fermentation of *A. tubingensis*

**Fig. 4:**
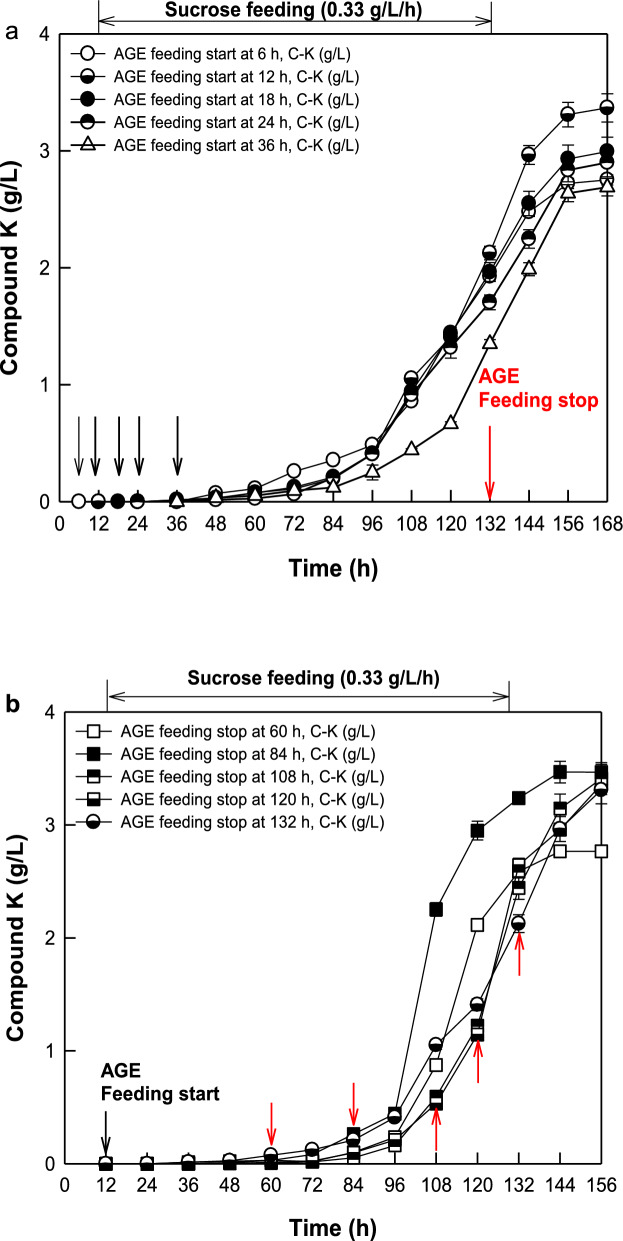
Effects of feeding-start and feeding-stop time points during continuous feeding of AGE on C-K production in fed-batch fermentation of *A. tubingensis.* **A** Effect of feeding-start time of AGE on C-K production. Feeding of AGE was stopped at 132 h. **B** Effect of feeding-stop time of AGE on C-K production. Feeding of AGE was started at 12 h. AGE at 16 g/L was continuously added. Sucrose at 20 g/L was initially added, and 40 g/L sucrose was continuously added from 12 to 132 h at a flow rate of 0.33 g/L/h. Black and red arrows indicate the feeding-start and feeding-stop time points of AGE, respectively

To determine the optimal feeding period of AGE as a reactant with a total added concentration of 16 g/L for the maximal production of C-K, the feeding-start and feeding-stop time points were varied from 6 to 36 h at a feeding-stop time of 132 h and from 60 to 132 h at a feeding-start time of 12 h, respectively. The highest concentration and productivity of C-K were observed during the period from the feeding start time 12 h (Fig. 4a) to the feeding-stop time 84 h (Fig. 4b) among the feeding-start and feeding-stop time points tested. As the optimal feeding period was used in fed-batch fermentation, the concentration, productivity, and molar conversion of C-K from PPD-type ginsenosides were 3.47 g/L, 24.1 mg/L/h, and 90.4%, respectively, which were 1.3-, 1.4-, and 1.3-fold higher than those of the feeding period from 36 to 132 h before optimization (Table 2).

**Table 2:** Effect of feeding period during continuous feeding of American ginseng extract (age) in fed-batch fermentation on the concentration, yields, productivity, and molar conversion of C-K for the biotransformation of AGE into C-K

No	F_start_ (h)	F_stop_ (h)	C-K_max_ (g/L)	*Y*_P/S_ (g/g)	*Y*_P/AGE_ (g/g)	q_P_ (mg/L/h)	Molar conversion (%)	*f*_t_ (h)
1	6	132	2.72 ± 0.02	0.05 ± 0.001	0.17 ± 0.001	17.4 ± 0.14	70.8 ± 0.57	156
2	12	132	3.31 ± 0.06	0.06 ± 0.001	0.20 ± 0.004	21.2 ± 0.38	86.2 ± 1.56	156
3	18	132	2.93 ± 0.05	0.05 ± 0.001	0.18 ± 0.003	18.8 ± 0.32	76.3 ± 1.29	156
4	24	132	2.83 ± 0.05	0.05 ± 0.001	0.18 ± 0.003	18.2 ± 0.32	73.7 ± 1.29	156
5	36	132	2.63 ± 0.04	0.04 ± 0.004	0.16 ± 0.002	16.9 ± 0.23	68.5 ± 0.95	156
6	12	60	2.77 ± 0.03	0.05 ± 0.001	0.17 ± 0.002	19.2 ± 0.18	72.1 ± 0.68	144
7	12	84	3.47 ± 0.05	0.06 ± 0.001	0.22 ± 0.003	24.1 ± 0.33	90.4 ± 1.24	144
8	12	108	3.41 ± 0.07	0.06 ± 0.001	0.21 ± 0.004	21.8 ± 0.45	88.8 ± 1.83	156
9	12	120	3.35 ± 0.02	0.06 ± 0.001	0.21 ± 0.001	21.4 ± 0.13	87.2 ± 0.53	156
10	12	132	3.31 ± 0.06	0.06 ± 0.001	0.20 ± 0.004	21.2 ± 0.39	86.2 ± 1.59	156

F_start_: feeding-start time, F_stop_: feeding-stop time

The effect of the total added concentration of AGE on C-K production in the fed-batch fermentation was investigated by varying the concentration from 8 to 32 g/L (Table 3). As the total added concentration of AGE was 8 g/L, the molar conversion of C-K from PPD-type ginsenosides in AGE was 100%. Above 8 g/L of total added AGE, the molar conversion decreased with increasing AGE concentration. However, the concentration (3.94 g/L) and productivity (27.4 mg/L/h) of C-K were highest at the total added concentration of 20 g/L AGE. The optimal conditions for continuous feeding of AGE for maximal biotransformation and production of C-K were 8 and 20 g/L of total added AGE during the feeding period from 12 to 84 h at flow rates of 0.11 and 0.28 g/L/h, respectively.

**Table 3:** Effect of total added concentration of age on C-K production in fed-batch fermentation of *A. tubingensis*

AGE (g/L)	C-K_max_ (g/L)	*Y*_P/S_ (g/g)	*Y*_P/AGE_ (g/g)	q_P_ (mg/L/h)	Molar conversion (%)	*f*_t_ (h)
8	1.91 ± 0.00	0.03 ± 0.000	0.24 ± 0.000	13.2 ± 0.00	100 ± 0.0	144
12	2.69 ± 0.08	0.04 ± 0.001	0.22 ± 0.006	18.6 ± 0.52	93.4 ± 2.6	144
16	3.47 ± 0.03	0.06 ± 0.001	0.22 ± 0.002	24.1 ± 0.21	90.4 ± 0.8	144
20	3.94 ± 0.01	0.07 ± 0.002	0.20 ± 0.001	27.4 ± 0.07	82.1 ± 0.2	144
24	3.56 ± 0.03	0.06 ± 0.001	0.15 ± 0.001	22.8 ± 0.16	61.8 ± 0.4	156
32	3.54 ± 0.02	0.06 ± 0.001	0.11 ± 0.001	21.1 ± 0.12	46.1 ± 0.3	168

### C-K production from PPD-type ginsenosides in age in fed-batch fermentation under optimized conditions for maximal biotransformation and production

**Fig. 5:**
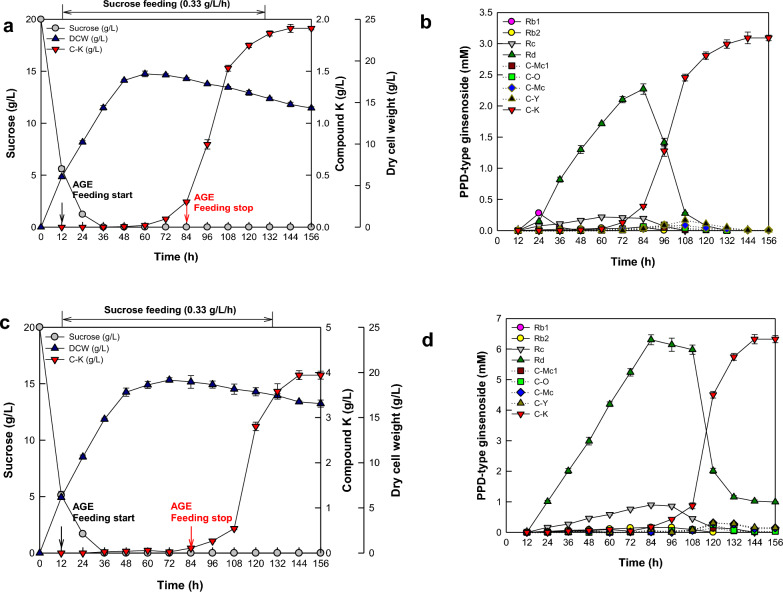
Maximal biotransformation and production of C-K from PPD-type ginsenosides in AGE in fed-batch fermentation of *A. tubingensis*. **A** Maximal biotransformation of C-K from PPD-type ginsenosides in 8 g/L AGE. **B** Concentration change of PPD-type ginsenosides in 8 g/L AGE during C-K production. AGE at 8 g/L was continuously added from 12 to 84 h at a flow rate of 0.11 g/L/h. **C** Maximal production of C-K from PPD-type ginsenosides in 20 g/L AGE. **D** Concentration change of PPD-type ginsenosides in 20 g/L AGE during C-K production. AGE at 20 g/L was continuously added from 12 to 84 h at a flow rate of 0.28 g/L/h. Sucrose at 20 g/L was initially added, and 40 g/L sucrose was then continuously added from 12 to 132 h at a flow rate of 0.33 g/L/h. Black and red arrows indicate the feeding-start and feeding-stop time points of AGE, respectively

Under the optimized conditions for the maximal biotransformation of C-K, *A. tubingensis* with a finial dry cell weight of 14.8 g/L produced 1.91 g/L (3.08 mM) C-K from 3.08 mM PPD-type ginsenoside in 8 g/L AGE for 144 h, with a productivity of 13.2 mg/L/h and a molar conversion of 100% (Fig. 5a). The molar conversion increased 3.0-fold compared to that of the fermentation with the initial 20 g/L sucrose in batch fermentation before feed optimization. C-K is produced from ginsenosides Rb1, Rb2, Rc, and Rd in ginseng extract by enzymes from *A. tubingensis* via the transformation pathways of Rb1 → Rd → F2 → C-K, Rb2 → C-O or Rd → C-Y or F2 → C-K, and Rc → C-Mc1 or Rd → C-Mc or F2 → C-K (Kim et al. 2021). Rb1, Rb2, and Rc in 8 g/L AGE supplied from 12 to 84 h of fermentation time were first converted to the intermediate Rd, which was accumulated up to 2.3 mM at 84 h with Rd supplied in 8 g/L AGE, and all PPD ginsenosides were completely converted to C-K after 144 h of fermentation time (Fig. 5b).

Under the optimized conditions for the maximal production of C-K, *A. tubingensis* with a final dry cell weight of 16.7 g/L converted 7.7 mM PPD-type ginsenosides in 20 g/L AGE into C-K for 144 h, with a concentration of 3.94 g/L (6.32 mM), a productivity of 27.4 mg/L/h, and a molar conversion of 82.1% (Fig. 5c), which were 3.1-, 3.1-, and 2.4-fold higher than those before feed optimization, respectively, and 2.1- and 2.1-fold higher and 17.9% lower than those using total added AGE of 8 g/L, respectively. In the fermentation, Rd was accumulated up to 6.3 mM (5.97 g/L) at 84 h. After 84 h, the accumulated Rd decreased by biotransformation to C-K with increasing fermentation time. However, after 132 h of fermentation, approximately 1 mM of Rd remained (Fig. 5d). To reduce the accumulation of Rd, the addition of highly active Rd-hydrolyzing glucosidase to the broth or transformation of its gene into *A. tubingensis* can be suggested. After 156 h of fermentation, the residual ginsenoside concentrations followed the order Rd (0.99 mM) > C-Mc (0.14 mM) > C-Y (0.11 mM) > C-Mc1 (0.03 mM) > C-O (0.03 mM).

### Purification from fermentation broth to food- and pharmaceutical-grade C-K via ethanol and resin treatments

Fermentation broth containing 1.91 g/L (3.08 mM) C-K obtained from 8 g/L AGE after 144 h of fermentation time (Fig. 5a) was used for C-K purification because other PPD-type ginsenosides in the broth contained less than 0.01 mM (Fig. 5b). The mycelium was extracted thrice, and the dried supernatant was extracted once with ethanol. All extracts were collected and ultrafiltered using Centricon. After ultrafiltration, 1.09 g of food-grade C-K was obtained with a purity of 58.9% and a recovery of 56.8%, as determined by HPLC (Table 4). The HPLC profile of AGE showed not only PPD-type ginsenosides but also unknown compounds (Additional file 1: Fig. S2a). The fermentation broth contained the unknown AGE-derived compounds (Additional file 1: Fig. S2b), and its HPLC profile was almost the same as that of ethanol and filter-treated C-K (Additional file 1: Fig. S2c), suggesting that the ethanol and filter treatments removed the cell debris, but not the AGE-derived unknown compounds. Ethanol and filter-treated C-K can be applied as food-grade C-K to the food industry because *A. tubingensis* is a GRAS fungus. Food-grade C-K was easily and economically prepared because this purification was performed using only ethanol and filter treatments.

**Table 4:** Purification of C-K from fermentation broth via ethanol and resin treatments

Step	Volume (L)	Dried solid (g)	C-K (g)	C-K concentration (g/L)	Purity (%)	Yield (%)	Recovery (%)
Fermentation broth	1.00			1.92 ± 0.00			100
1^st^ Ethanol extraction	1.00	2.35 ± 0.02	0.87 ± 0.01	0.87 ± 0.01	37.0 ± 0.11	45.3 ± 0.52	45.3 ± 0.52
2^nd^ Ethanol extraction	1.00	0.98 ± 0.01	0.34 ± 0.01	0.34 ± 0.01	34.7 ± 0.16	32.4 ± 0.95	63.0 ± 1.76
3^rd^ Ethanol extraction	1.00	0.60 ± 0.01	0.13 ± 0.02	0.13 ± 0.01	21.7 ± 1.23	18.3 ± 2.82	69.8 ± 3.12
Supernatant extraction	1.00	0.31 ± 0.02	0.08 ± 0.01	0.08 ± 0.01	25.8 ± 0.05	4.17 ± 0.52	74.0 ± 1.74
All extraction	0.20	4.24 ± 0.05	1.42 ± 0.01	7.10 ± 0.05	33.5 ± 0.65	74.0 ± 0.52	74.0 ± 0.52
Ultrafiltration	0.05	1.85 ± 0.03	1.09 ± 0.01	218 ± 6.00	58.9 ± 0.43	76.8 ± 0.70	56.8 ± 0.56
Octadecyl-silica resin	0.03	1.21 ± 0.03	0.85 ± 0.01	28.3 ± 0.33	70.2 ± 1.08	77.9 ± 0.92	44.3 ± 0.64
C18 resin in prep-HPLC	0.01	0.50 ± 0.01	0.48 ± 0.01	48.0 ± 1.10	96.0 ± 0.04	56.4 ± 1.18	25.0 ± 0.59

The ultrafiltered C-K was further purified by treatment with an ODS A resin, which removed most of the AGE-derived unknown compounds (Additional file 1: Fig. S2d), and a hydrosphere C18 resin in prep-HPLC, which isolated C-K as a single peak (Additional file 1: Fig. S2E). The HPLC profile of resin-treated C-K was almost same as that of standard C-K (≥ 98% purity) (Additional file 1: Fig. S2f). After the treatments of two resins, 0.48 g of C-K was obtained with a purity of 96.0% and a recovery of 25.0%, as determined by HPLC. This purity indicates that the purified C-K can be used as pharmaceutical-grade C-K to the pharmaceutical industry. To the best of our knowledge, this is the first report on the purification of C-K from the fermentation broth.

## Discussion

Although filamentous fungi require a carbon source to secrete hydrolytic enzymes, a high concentration of carbon source results in a decrease in hydrolytic enzymes via catabolic repression (Dos Reis et al. 2013). The inhibition of enzymatic reactions caused by high concentrations of substrate is reduced by the fed-batch fermentation process (Qu et al. 2013). In the process, it is important to maintain a low concentration of carbon source during fermentation (Rohman et al. 2022). Fed-batch fermentation of reactant as a precursor for biotransformation has also been used because of its inhibition of cell growth at high concentrations (Gomes et al. 2012; Park et al. 2006). Ginseng extract as a precursor was added to the fermentation broth in the stationary phase because it inhibits cell growth (Song et al. 2022; Zhou et al. 2008). To overcome the inhibition of the carbon source sucrose and the precursor ginseng extract at high concentrations, we performed fed-batch fermentation and optimized the feed type, concentration, and period for sucrose and ginseng extract in fed-batch fermentation. After the optimization of feed type, concentration, and period for sucrose and, the concentration (3.94 g/L) and productivity (27.4 mg/L/h) of C-K increased 3.1-fold compared to those (1.29 g/l and 8.96 mg/L/h) in batch fermentation.

Bacteria and fungi have been used to produce C-K by fermentation, and the final concentration and productivity of C-K by fungi is higher than those by bacteria (Chi et al. 2006; Shin and Oh 2016). Although the comparison was not exact because the different type and concentration of ginseng extract were used for C-K production, the production of C-K from PPD-type ginsenosides in ginseng extracts by fermentation using fungi is summarized in Table 5. The same strain *A. tubingensis* KCTC 14,166 produced 2.47 g/L C-K by AGE feeding in flask fermentation for 144 h, with a productivity of 17.1 mg/L/h, which were the previously highest concentration and productivity (Song et al. 2022). Among other fungi, *Paecilomyces bainier* sp. 299 exhibited the highest concentration (1.25 g/L) and productivity (8.6 mg/L/h) of C-K in a fermenter. The concentration (3.94 g/L) and productivity (27.4 mg/L/h) of C-K in fed-batch fermentation using a fermenter by *A. tubingensis* KCTC 14,166 were 3.2-fold higher than those of *P. bainier* sp. 299, respectively, and they were 98- and 43-fold higher than those of *Aspergillus niger* FMBS 494, respectively, which were the previously reported highest values (0.04 g/L and 0.625 mg/L/h) among GRAS fungi (Li and Ji 2017). These results indicate that feed optimization in fed-batch fermentation is an effective tool for increasing C-K production by reducing the inhibition of the formation of C-K-producing enzymes at high concentrations of sucrose and the toxicity of high concentrations of ginseng extract to cells.

**Table 5:** Production of C-K from protopanaxadiol (PPD)-type ginsenosides in ginseng extract via fermentation using fungi

Fungus	Ginseng extract	C-K (g/L)	Molar conversion (%)	Productivity (mg/L/h)	Fermentation system	References
*Fusarium sacchari*	Saponins of *Panax notoginseng* extract	0.25	24	1.74	Flask	Han et al. (2007)
*Paecilomyces bainier* sp. 229	Saponins of *Panax notoginseng* leaves	1.25	82.6	8.6	Fermenter	Zhou et al. (2008)
*Ganoderma lucidum* CRC 37066	American ginseng root extraction residue	0.005	3.0	0.01	Flask	Hsu et al. (2013)
*Aspergillus niger* KACC 46494 *Aspergillus oryzae* KACC 40247	Korean ginseng berry extract	NC NC	10.3 3.4	NC NC	Flask	Li et al. (2016)
*Aspergillus niger* FMBS 494	Korean ginseng	0.04	66.8	0.625	Flask	Li and Ji (2017)
*Cordyceps sinensis*	Red ginseng extract	0.11	NC	NC	Fermenter	Bae et al. (2011)
*Aspergillus tubingensis* KCTC 14166	American ginseng extract	1.87 2.47	86.4 64.3	12.9 17.1	Flask	Song et al. (2022)
*Aspergillus tubingensis* KCTC 14166	American ginseng extract	1.91 3.94	100 82.1	13.2 27.4	Fermenter	This study

*NC* Not calculated

In conclusion, for the increased biotransformation of ginseng extract into C-K, the feeding conditions of the carbon source sucrose and reactant AGE were optimized in fed-batch fermentation. Feed optimization resulted in the enhanced conversion of American ginseng extract into C-K by reducing the inhibition of sucrose for the formation of C-K-producing enzyme as well as the toxicity of ginseng extract to cells. Moreover, 59% food-grade C-K was prepared by only ethanol and filter treatments, and 96% pharmaceutical-grade C-K was prepared by ethanol, filter, and resin treatments. To our knowledge, this is the first trial of fed-batch fermentation to convert ginseng extract into deglycosylated ginsenoside, the highest concentration and productivity of C-K produced via fermentation, the first fermentation to completely convert all PPD-type ginsenosides to C-K, and the first report on the purification of C-K from fermentation broth. This feeding strategy is a novel and economical solution to improve the biotransformation of ginseng extract into C-K via fermentation and may also contribute to the improved biotransformation of other chemicals via fermentation.

## Supplementary Information

**Additional file 1: Figure S1.** Comparison of pulse and continuous feedings of the American ginseng extractin fed-batch fermentation of *Aspergillus tubingensis*. For pulse feeding, AGE at 8 g/l was intermittently added twice at 36 and 48 h. For continuous feeding, AGE at 16 g/l was added at a flow rate of 0.167 g/l/h from 36 to 132 h. Sucrose at 20 g/l was initially added, followed by the continuous addition of 40 g/l sucrose from 12 to 132 h at a flow rate of 0.33 g/l/h. Black and red arrows represent the feeding-start and feeding-stop time points of AGE, respectively. Pink arrows indicate pulse feeding. **Figure S2.** High-performance liquid chromatographyprofiles of ginsenosides in AGE, compound Kin the fermentation broth, ethanol and filter-treated C-K, octadecyl-silicaA resin-treated C-K, C18 resin-treated C-K, and standard C-K.HPLC profile of ginsenosides in AGE.HPLC profile of C-K in the fermentation broth.HPLC profiles of food-grade C-K obtained from ethanol and filter treatments.HPLC profile of ODS A resin-treated C-K.HPLC profile of pharmaceutical-grade C-K obtained from treatment with C18 resin using preparative high-performance liquid chromatography.HPLC profile of standard C-K.

## Data Availability

The data obtained for all analyses conducted in this study are presented in the article.
