# Supplementary material for: Production of ginsenoside compound K from American ginseng extract by fed-batch culture of Aspergillus tubingensis
Source: AMB Express. 2023 Jun 25;13:64. doi: 10.1186/s13568-023-01556-5 (PMC10290978; doi:10.1186/s13568-023-01556-5)
Supplement: Supplementary file 1 — Additional file 1: Figure S1. Comparison of pulse and continuous feedings of the American ginseng extractin fed-batch fermentation of Aspergillus tubingensis. For pulse feeding, AGE at 8 g/l was intermittently added twice at 36 and 48 h. For continuous feeding, AGE at 16 g/l was added at a flow rate of 0.167 g/l/h from 36 to 132 h. Sucrose at 20 g/l was initially added, followed by the continuous addition of 40 g/l sucrose from 12 to 132 h at a flow rate of 0.33 g/l/h. Black and red arrows represent the feeding-start and feeding-stop time points of AGE, respectively. Pink arrows indicate pulse feeding. Figure S2. High-performance liquid chromatographyprofiles of ginsenosides in AGE, compound Kin the fermentation broth, ethanol and filter-treated C-K, octadecyl-silicaA resin-treated C-K, C18 resin-treated C-K, and standard C-K.HPLC profile of ginsenosides in AGE.HPLC profile of C-K in the fermentation broth.HPLC profiles of food-grade C-K obtained from ethanol and filter treatments.HPLC profile of ODS A resin-treated C-K.HPLC profile of pharmaceutical-grade C-K obtained from treatment with C18 resin using preparative high-performance liquid chromatography.HPLC profile of standard C-K. [file 13568_2023_1556_MOESM1_ESM.pdf]

AMB Express

Supplementary data

Production of ginsenoside compound K from American ginseng extract by fed-batch culture of *Aspergillus tubingensis*

Woo-Seok Song, Kyung-Chul Shin, Deok-Kun Oh

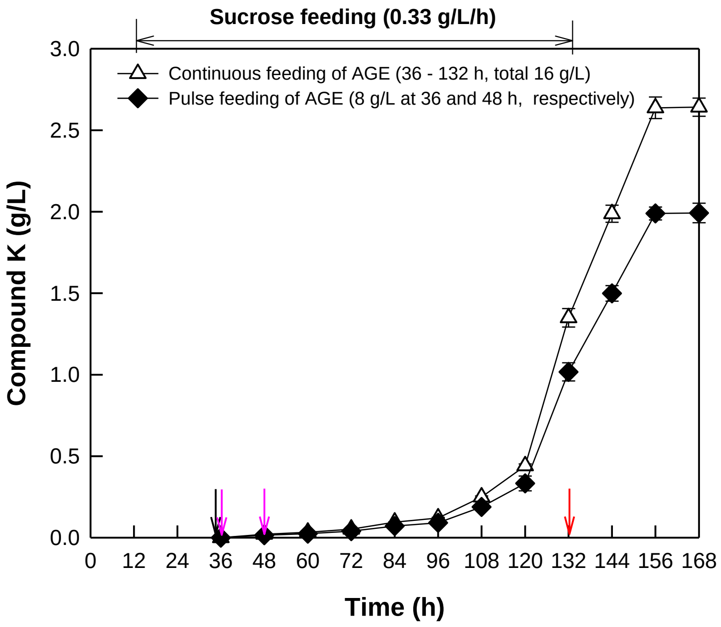

Fig. S1 Comparison of pulse and continuous feedings of the American ginseng extract (AGE) in fed-batch fermentation of *Aspergillus tubingensis*. For pulse feeding, AGE at 8 g/l was intermittently added twice at 36 and 48 h. For continuous feeding, AGE at 16 g/l was added at a flow rate of 0.167 g/l/h from 36 to 132 h. Sucrose at 20 g/l was initially added, followed by the continuous addition of 40 g/l sucrose from 12 to 132 h at a flow rate of 0.33 g/l/h. Black and red arrows represent the feeding-start and feeding-stop time points of AGE, respectively. Pink arrows indicate pulse feeding.

a

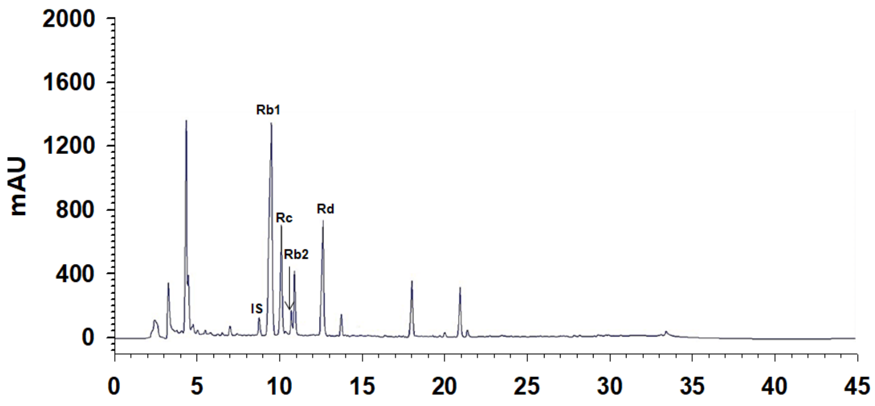

2000
1600
1200
800
400
0
mAU
Rb1
Rc
Rb2
Rd
IS
0
5
10
15
20
25
30
35
40
45

b

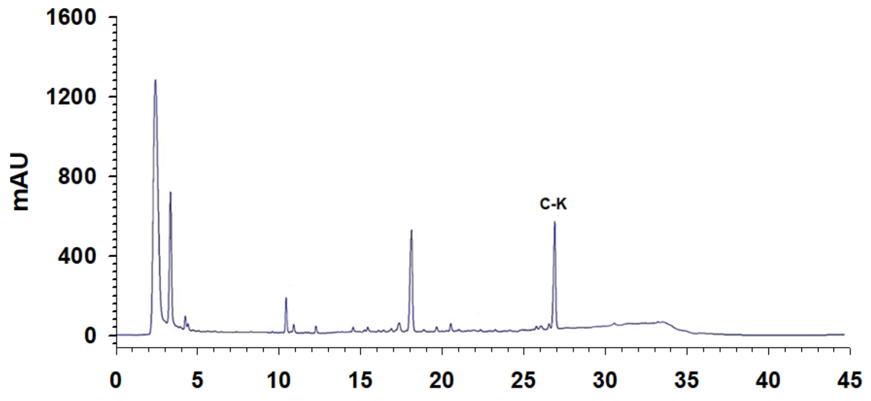

1600
1200
800
400
0
mAU
C-K
0
5
10
15
20
25
30
35
40
45

c

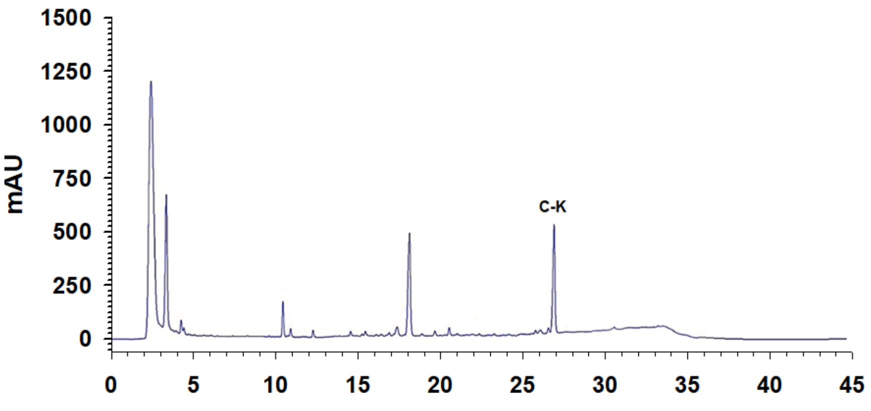

1500
1250
1000
750
500
250
0
mAU
C-K
0
5
10
15
20
25
30
35
40
45

d

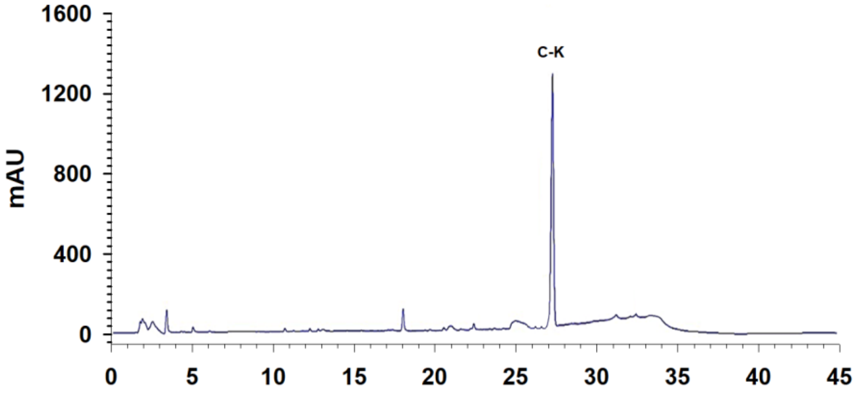

e

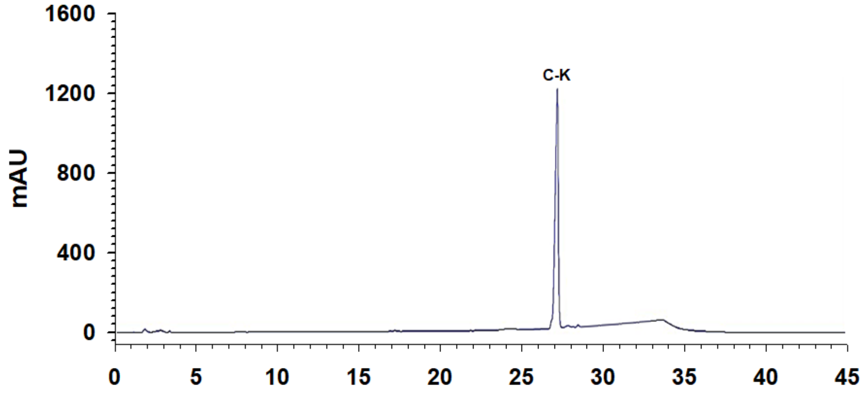

f

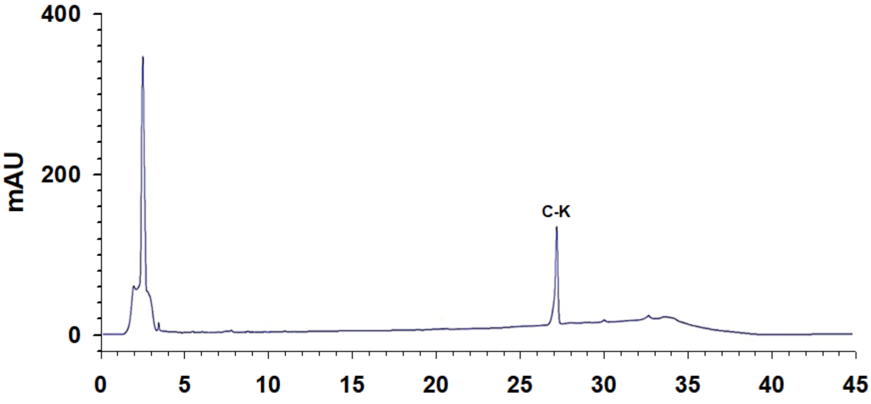

Fig. S2 High-performance liquid chromatography (HPLC) profiles of ginsenosides in AGE, compound K (C-K) in the fermentation broth, ethanol and filter-treated C-K, octadecyl-silica (ODS) A resin-treated C-K, C18 resin-treated C-K, and standard C-K.

(**a**) HPLC profile of ginsenosides in AGE. (**b**) HPLC profile of C-K in the fermentation broth. (**c**) HPLC profiles of food-grade C-K obtained from ethanol and filter treatments. (**d**) HPLC profile of ODS A resin-treated C-K. (**e**) HPLC profile of pharmaceutical-grade C-K obtained from treatment with C18 resin using preparative high-performance liquid chromatography (prep-HPLC). (**f**) HPLC profile of standard C-K.
